# Tumor-derived alpha fetoprotein directly impacts human natural killer cell activity and viability

**DOI:** 10.1186/2051-1426-3-S2-P289

**Published:** 2015-11-04

**Authors:** Lazar Vujanovic, Elizabeth Stahl, Angela Pardee, Simon Watkins, Gregory Gibson, Lisa H Butterfield

**Affiliations:** 1 University of Pittsburgh Cancer Institute, Pittsburgh, PA, USA; 2 Department of Cell Biology and Physiology, University of Pittsburgh School of Medicine, Pittsburgh, PA, USA; 3 University of Pittsburgh, Pittsburgh, PA, USA

## 

Alpha-fetoprotein (AFP) is an oncofetal antigen produced by hepatocellular carcinomas (HCC). Previous studies demonstrated that tumor-derived AFP (tAFP) is a glycoprotein that has an immunosuppressive role on natural killer (NK), T, B, and dendritic (DC) cells which may play a role in HCC pathogenesis. Defects in NK cells have been attributed to tAFP-mediated immunosuppression of DC. However, a direct tAFP effect on NK cells remains unexplored. Here we compared the ability of cord blood-derived AFP (nAFP) to that of tAFP to modulate human NK cell activity and longevity *in vitro*. Short-term exposure to tAFP and, especially, nAFP proteins induced a unique pro-inflammatory, IL-2 hyperresponsive phenotype in healthy donor NK cells as measured by CD69 upregulation, IL-1β, IL-6 and TNF secretion, and enhanced tumor cell killing. In contrast, extended co-culture with tAFP, but not nAFP, inhibited NK cell proliferation and viability. NK cell activation was directly mediated by the AFP protein itself, while their viability was affected by the low molecular mass cargo that co-purified with tAFP. Overall, these data show that nAFP and tAFP induce similar yet distinct changes in NK cell function and viability, respectively. Defining the impact of circulating AFP on NK cells may be crucial to understand the NK cell functional deficits described in HCC patients.

